# Structural investigations of the ferredoxin and terminal oxygenase components of the biphenyl 2,3-dioxygenase from *Sphingobium yanoikuyae *B1

**DOI:** 10.1186/1472-6807-7-10

**Published:** 2007-03-09

**Authors:** Daniel J Ferraro, Eric N Brown, Chi-Li Yu, Rebecca E Parales, David T Gibson, S Ramaswamy

**Affiliations:** 1 Department of Biochemistry, University of Iowa Roy J. and Lucille A. Carver College of Medicine, 51 Newton Road, 4-611 BSB, Iowa City, Iowa, 52242, USA; 2 Department of Microbiology, University of Iowa Roy J. and Lucille A. Carver College of Medicine, 51 Newton Road, 3-711 BSB, Iowa City, Iowa, 52242, USA; 3 Section of Microbiology, University of California, Davis, 226 Briggs Hall, One Shields Avenue, Davis, CA 95616, USA

## Abstract

**Background:**

The initial step involved in oxidative hydroxylation of monoaromatic and polyaromatic compounds by the microorganism *Sphingobium yanoikuyae *strain B1 (B1), previously known as *Sphingomonas yanoikuyae *strain B1 and *Beijerinckia *sp. strain B1, is performed by a set of multiple terminal Rieske non-heme iron oxygenases. These enzymes share a single electron donor system consisting of a reductase and a ferredoxin (BPDO-F_B1_). One of the terminal Rieske oxygenases, biphenyl 2,3-dioxygenase (BPDO-O_B1_), is responsible for B1's ability to dihydroxylate large aromatic compounds, such as chrysene and benzo[*a*]pyrene.

**Results:**

In this study, crystal structures of BPDO-O_B1 _in both native and biphenyl bound forms are described. Sequence and structural comparisons to other Rieske oxygenases show this enzyme to be most similar, with 43.5 % sequence identity, to naphthalene dioxygenase from *Pseudomonas *sp. strain NCIB 9816-4. While structurally similar to naphthalene 1,2-dioxygenase, the active site entrance is significantly larger than the entrance for naphthalene 1,2-dioxygenase. Differences in active site residues also allow the binding of large aromatic substrates. There are no major structural changes observed upon binding of the substrate. BPDO-F_B1 _has large sequence identity to other bacterial Rieske ferredoxins whose structures are known and demonstrates a high structural homology; however, differences in side chain composition and conformation around the Rieske cluster binding site are noted.

**Conclusion:**

This is the first structure of a Rieske oxygenase that oxidizes substrates with five aromatic rings to be reported. This ability to catalyze the oxidation of larger substrates is a result of both a larger entrance to the active site as well as the ability of the active site to accommodate larger substrates. While the biphenyl ferredoxin is structurally similar to other Rieske ferredoxins, there are distinct changes in the amino acids near the iron-sulfur cluster. Because this ferredoxin is used by multiple oxygenases present in the B1 organism, this ferredoxin-oxygenase system provides the structural platform to dissect the balance between promiscuity and selectivity in protein-protein electron transport systems.

## Background

*Sphingobium yanoikuyae *B1 (B1), previously known as *Sphingomonas yanoikuyae *B1 and *Beijerinckia *sp. strain B1 [1], was isolated by virtue of its ability to use biphenyl as its sole source of carbon and energy for growth [2]. B1 is capable of using naphthalene, phenanthrene, anthracene, toluene, *m*- and *p*-xylene as sole sources of carbon and energy [3]. Other compounds are also oxidized by this microorganism and many of these are converted to *cis*-dihydrodiols. B1 remains one of the only known microbes, along with *Mycobacterium vanbaalenii *PYR1 [4] and *Sphingomonas *sp. strain CHY-1 [5-7], able to oxidize large aromatic hydrocarbons such as benzo[*a*]pyrene, benzo[*a*]anthracene and chrysene [8] to *cis*-dihydrodiols.

In the B1 genome, at least six sets of putative oxygenase genes are present [9] and are all believed to share a common electron donor system [10]. The genes *bphA1A2f*, which encode BPDO-O_B1_, have been sequenced and found to encode a Rieske oxygenase (RO) [11-13] related to naphthalene and biphenyl dioxygenases [14]. BPDO-O_B1 _is responsible for the oxidation of large aromatic compounds, such as benzo[*a*]pyrene, by B1. Structural information [15-17] and molecular modeling [18] have been used to determine features important for substrate specificity in other biphenyl oxidizing oxygenases; however, most of the effort has been targeted at understanding how biphenyl dioxygenases catalyze the degradation of polychlorinated biphenyls (reviewed in [19-22]). Previous structural studies of several ROs provide insight into common features of how the terminal component of the RO systems are organized and how substrate interacts with the enzyme to form the hydroxylated product [23]. To date, structures of enzymes that catalyze *cis*-dihydroxylation of aromatic substrates with more than three rings have not been reported.

Rieske oxygenase systems have multiple components whose function is to transfer electrons from NAD(P)H to active molecular oxygen and ultimately oxidize aromatic hydrocarbon substrates [23]. A single reductase for the multiple Rieske oxygenases is present in the B1 genome [10]. This reductase passes one electron at a time from NAD(P)H to the Rieske ferredoxin, BPDO-F_B1_, which in turn passes the electron on to the dioxygenase enzyme [24]. The terminal oxygenase component is responsible for catalyzing the addition of molecular oxygen to the aromatic substrate. This occurs at the mononuclear iron, contained in a large, primarily hydrophobic active site. The residues that form the active site have been shown to control substrate specificity.

Here we report the structures of BPDO-F_B1 _and BPDO-O_B1_. The structure of BPDO-F_B1 _shows similarities and important differences compared to other known Rieske dioxygenase ferredoxins. Structures of BPDO-O_B1_, in both the native form and bound to biphenyl, are presented. These structures demonstrate how BPDO-O_B1 _binds substrate in the active site. We also discuss the similarities and differences of this terminal oxygenase to other RO terminal oxygenase structures that have been previously determined and how these differences play a role in substrate specificity and regio- and stereoselectivity of product formation.

## Results & discussion

### Ferredoxin structure determination

The asymmetric unit contains two copies of the BPDO-F_B1 _molecule. The final model contains residues 3 – 104 in chains A and B. The structure has been refined to a resolution of 1.9 Å with a final R_factor _of 19.3% and an R_free _of 24.0%. The first two N-terminal and last three C-terminal residues could not be modeled into the electron density. The superposition of all Cα atoms in both chains using Lsqman [25] had an RMSD of 0.40 Å. Residues Lys-25, Pro-104, and Glu-95 through Gly-97 had the largest deviations between chains A and B. The surface loop containing Lys-25 and Met-26 had little to no density for their side chains, Pro-104 is the last residue modeled, while Asp-96 assumes two conformations in chain B. The Rieske [2Fe-2S] cluster had isotropic displacement factors of 19.6 and 18.5 Å^2 ^for chains A and B respectively. Crystallographic statistics are reported in Table 1.

### Oxygenase structure determination

The asymmetric unit contains the entire BPDO-O_B1_α_3_β_3 _hexamer and residues 6 – 454 of the α subunits and residues 5 – 174 of the β subunits were modeled into the electron density. The structure has been refined to a resolution of 1.7 Å with a final R_factor _of 18.8 % and an R_free _of 22.9 %. The loop region located at the entrance of the α subunit active site is disordered, with higher than average B-factors and low side-chain electron density. This region spanned from residues 220 – 240 with residues 235 – 239 being the most disordered. Corresponding regions in other ROs are also disordered [15,26-30]. Lsqkab [25] was used to determine the superposition RMSDs of the three α and β subunits in the asymmetric unit. A few regions in the α subunit, residues 108 – 123 and 411 – 434, and in the β subunit, residues 77 – 85 and 139 – 148, had higher than average RMSDs when compared; however, electron density clearly defined the coordinates of these residues. Crystallographic statistics are reported in Table 1.

### Structure of the substrate-free oxygenase enzyme

**Figure 1:**
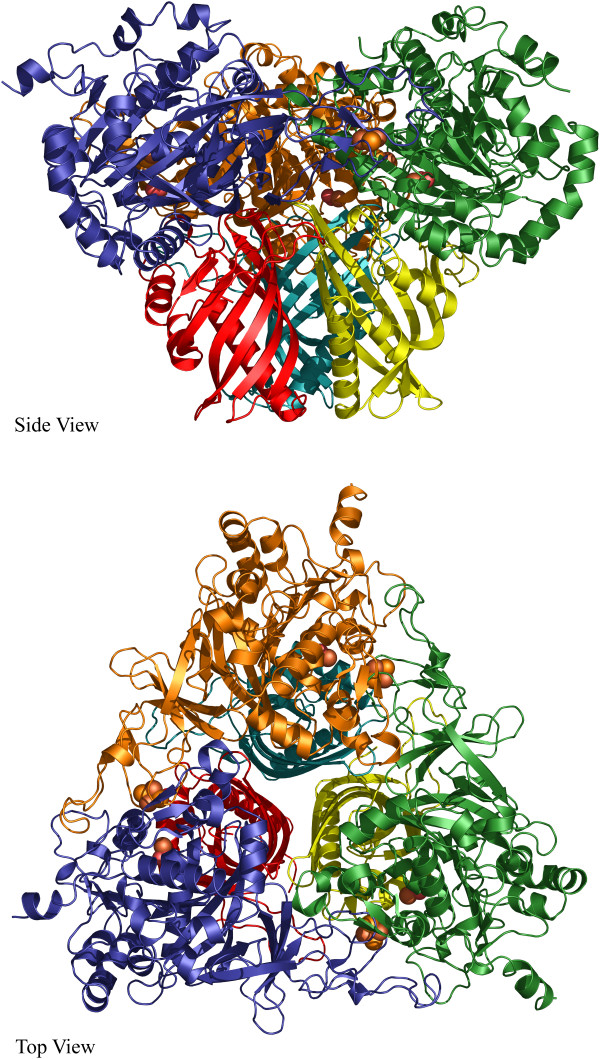
**Top and side view of the BPDO-O_B1 _hexamer**. The structure of biphenyl 2,3-dioxygenase is a mushroom-shaped α_3_β_3 _hexamer. This quaternary structure is typical of αβ Rieske oxygenases. This structure allows a mononuclear iron from one α subunit to come within ~15 Å of a Rieske cluster from a neighboring subunit, allowing electron transfer to take place.

The three copies of the α and β subunits form an α_3_β_3 _quaternary structure similar to the quaternary structure observed in all known RO structures. The hexamer forms a mushroom-shaped structure, illustrated in Figure 1, where the three α subunits form the cap and the three β subunits form the stem. The hexamer structure is believed to be the active biological unit, similar to other known ROs.

The α subunit consists of 454 residues, which form two domains. The N-terminal portion consists of a Rieske [2Fe-2S] iron-sulfur cluster domain, defined by residues 40 – 140. The Rieske domain consists of β strands and loops, forming an ISP domain fold [31]. Four residues, two histidines and two cysteins, in the α subunit coordinate the Rieske non-heme iron cluster [2Fe-2S]. His-82 and His-103 coordinate one iron, while Cys-80 and Cys-100 coordinate the other. The C-terminal domain is a mix of helices and strands forming a TBP-like or helix-grip fold and is a member of the Bet v1-like superfamily [31]. The structural conservation suggests that the intraprotein electron transport in BPDO-O_B1 _is similar to the system described for NDO-O_9816-4 _and benzoate dioxygenase [32-37].

**Figure 2:**
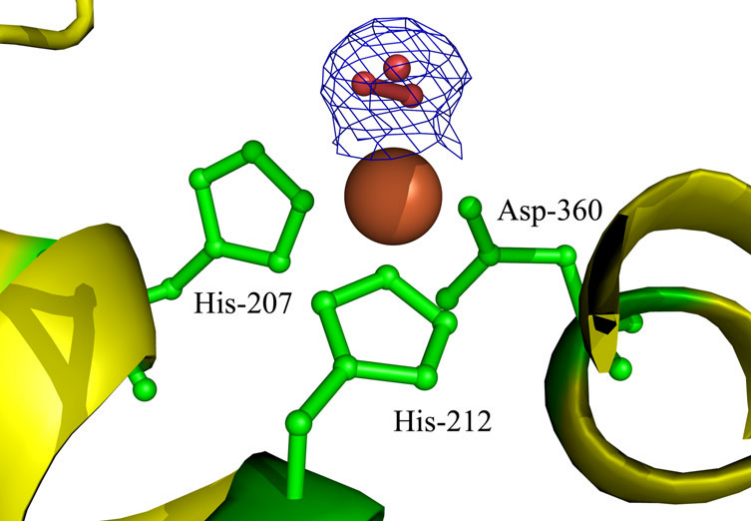
**Mononuclear iron bound to oxygen and water**. Active site of BPDO-O_B1 _showing the dioxygen molecule and water molecule modeled. The dioxygen molecule is shown as a ball and stick molecule, while a sphere represents the water molecule. The mesh is 1.0 σ 2Fo-Fc electron density.

The mononuclear iron is coordinated by two histidines, His-207 and His-212, and one aspartate, Asp-360, at the rear of the active site. This iron has been shown to bind water(s) or dioxygen in other structures. In BPDO-O_B1_, a clear bimodal electron density distribution was not observed, but instead an egg-shaped electron density above the mononuclear iron was present (Figure 2). When a single water/hydroxide molecule was modeled in this position, it resulted in residual positive density on the Fo-Fc electron density maps. Residual positive electron density was not observed on the Fo-Fc electron density maps when modeling molecular oxygen as the fourth iron ligand; however, refinement with relaxed restraints (refinement restraints allowing the oxygen-oxygen bond an RMSD of 0.3Å) placed the oxygen-oxygen bond distance at 0.8 – 1.1Å in the three subunits. This is slightly shorter than the average distance of 1.21 Å between two oxygen atoms in O_2_. B-factors for the refined oxygen molecule also suggested that it was less than fully occupied. The final model has both molecular oxygen and water bound to the iron in partially occupied states (Figure 2). In previous studies, both dioxygen species [15,27,38,39] and water [16,26,28,29,39-42] have been observed as ligands in RO crystals grown in atmospheric conditions. The BPDO-O_B1 _crystals were grown in atmospheric conditions, therefore molecular oxygen or water could constitute the fourth ligand on the mononuclear iron.

The β subunit of BPDO-O_B1 _has a Cystatin-like protein fold and belongs to the protein superfamily of NTF2-like proteins [31]. The function of the β subunits of ROs is not well understood and reports vary as to whether or not mutations in the β subunit of ROs can influence regioselectivity of product formation (reviewed in [43]). In the case of BPDO-O_B1_, the β subunit is not directly involved in creating the topology of the active site; however, the mononuclear iron is within 11 Å of the α-β subunit interface. This distance may allow select β-subunit side chains to indirectly affect the topology of the active site by interacting with residues that directly form the active site.

### Structure of the biphenyl bound oxygenase enzyme

**Figure 3:**
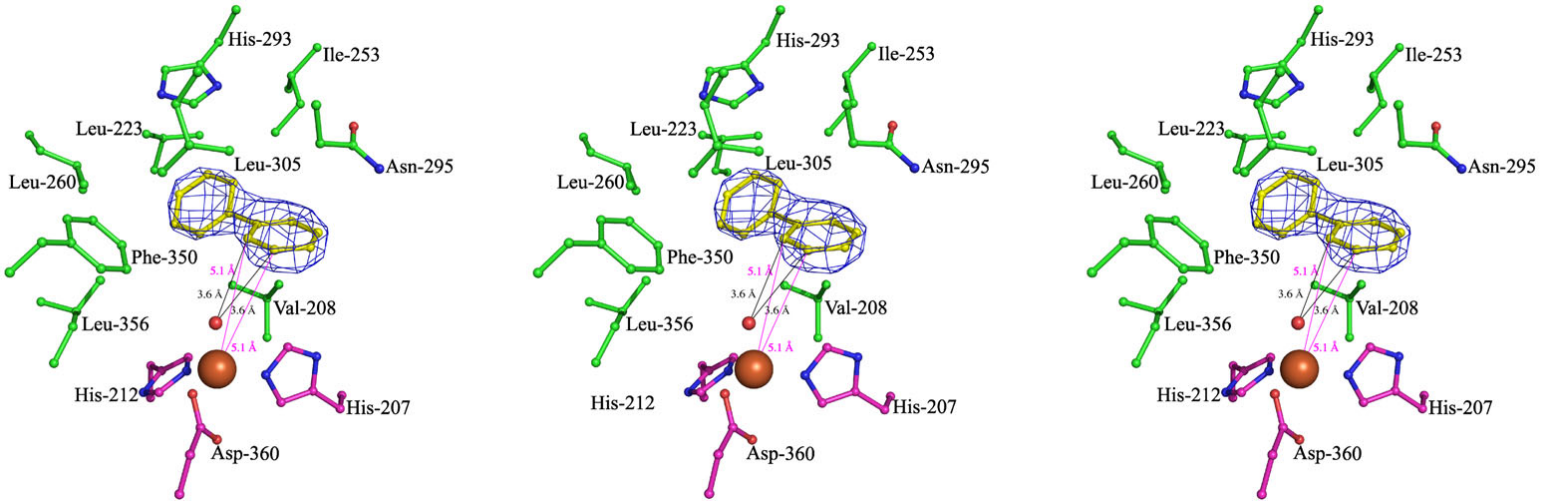
**BPDO-O_B1 _bound to biphenyl**. Stereo image of the active site of BPDO-O_B1 _with biphenyl bound. The biphenyl molecule is shown in yellow. A 1.0 σ 2Fo-Fc electron density map calculated without biphenyl present (unbiased) is shown in blue mesh for the biphenyl ligand. The residues that coordinate the mononuclear iron are shown in red, while the residues that form the active site pocket are shown in green. Black lines show distances between the biphenyl carbons and the mononuclear iron and red lines show distances between the biphenyl carbons and the water/hydroxide bound to the mononuclear iron. Left and center images are wall-eyed stereo, center and right images are cross-eyed stereo.

B1 has been shown to catalyze the dihydroxylation of biphenyl at positions 2 and 3 of the carbon ring [2,44] and recent studies have confirmed that BPDO-O_B1 _is responsible for this activity [45]. Previous structural investigations of ROs have demonstrated that substrate bound in the active site is oriented such that the carbon(s) oxidized in the dihydroxylation reaction is (are) closest to the mononuclear iron [16,26,27,30,40,42,46]. This trend is also seen in the structure of BPDO-O_B1 _bound to biphenyl. The 2 and 3 carbons of biphenyl are positioned closest to the iron, with a water/hydroxide molecule bound to the iron positioned between the substrate and the iron (Figure 3). Crystals were grown and biphenyl was added in the presence of atmospheric oxygen. The enzyme was oxidized and no electron source was added; therefore, catalysis did not occur. We are unable to determine whether the iron is coordinated to a water molecule (hydroxide ion) or an oxygen molecule at this resolution. No significant changes in the active site main chain were observed near the mononuclear iron compared to the native structure. However, small changes in the main-chain and side-chain positions of the distal portion of the active site were observed. The largest changes were seen in the loop regions that cover the entrance to the active site. The loop main-chain was pushed slightly away (~0.2–0.3 Å) from the substrate in the complex structure, while the main-chain proximal to the mononuclear iron remained static.

### Comparison of BPDO-F_B1 _to other Rieske oxygenase ferredoxins

**Figure 4:**
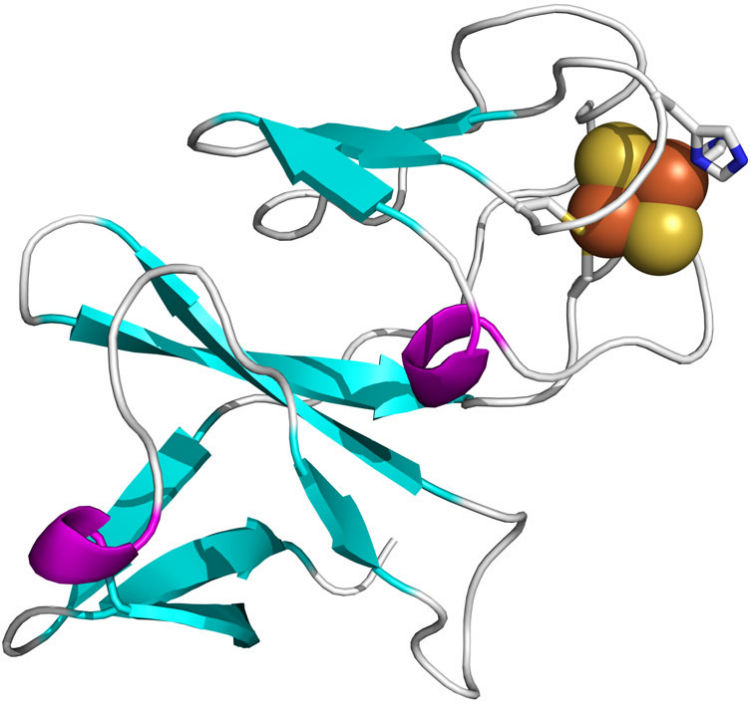
**Ribbon diagram of BPDO-F_B1 _showing the location of the Rieske iron-sulfur cluster**. The ribbon diagram shows the conserved Rieske binding domain of the ferredoxin, where the Rieske cluster is positioned near the surface of the protein. The Rieske cluster is shown as spheres, where iron is orange and sulfur is yellow. The residues that directly interact with the Rieske cluster are shown as sticks. Beta sheets are shown in blue and alpha helices are shown in purple.

The structure of the biphenyl dioxygenase system's ferredoxin component, BPDO-F_B1_, is the fourth Rieske oxygenase ferredoxin structure to be determined and shares significant sequence and structural homology with these proteins (Table 2). BPDO-F_B1 _has structural features similar to other ferredoxins including three stacked beta sheets and a solvent exposed Rieske cluster (Figure 4) [47]. The protein fold surrounding the Rieske cluster is similar in all Rieske ferredoxins, including the high-potential Rieske ferredoxins found in respiratory electron transport chains such as the bc_1 _complex [48] and the Rieske clusters found in dioxygenase enzymes such as BPDO-O_B1_.

**Figure 5:**
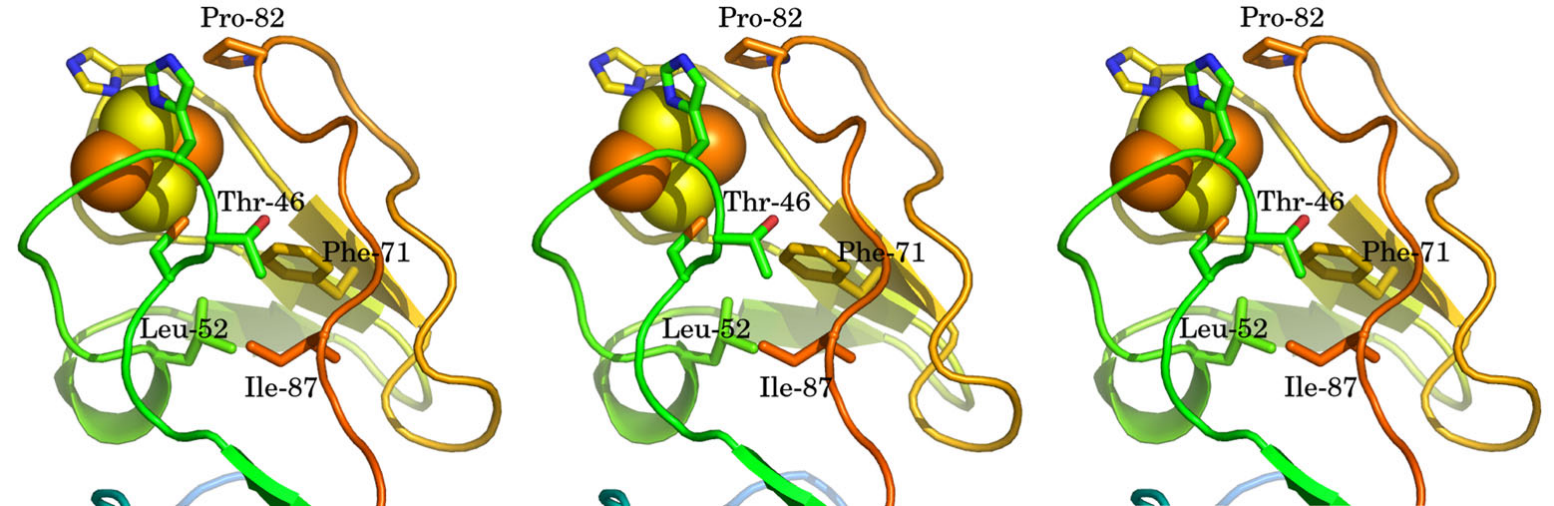
**Stereo images showing the conserved residues near the Rieske cluster of BPDO-F_B1_**. The cartoon diagram shows that side chains of Thr-46, Leu-52, Phe-71, and Ile-87 form a highly conserved core among the Rieske oxygenase ferredoxins near the iron-sulfur cluster. The residues flanking Pro-82 are proline in BPDO-F_LB400 _and some high-potential Rieske ferredoxins, but are not proline in BPDO-F_B1_. Left and center images are wall-eyed stereo, center and right images are cross-eyed stereo.

In addition to the conserved CXH-CXXH motif present in all Rieske ferredoxins, there are two additional conserved residues, Phe-71 and Pro-82, in the dioxygenase ferredoxins [47,49,50]. The phenylalanine is part of the small ferredoxin core near the Rieske cluster. The neighboring side-chains include Thr-46, Leu-52 and Ile-87. These residues are highly conserved with Thr-46 most commonly being a serine or a threonine, Leu-52 existing almost exclusively as a leucine, and Ile-87 being the most varied with leucine as the most common substitution (Table 3). The conserved proline is located at a hairpin turn at the apex of the ferredoxin and has previously been classified as part of a polyproline loop in Rieske ferredoxins (Figure 5). Unlike the biphenyl ferredoxin from LB400, which has three consecutive prolines, this ferredoxin has only a single proline in the loop.

**Figure 6:**
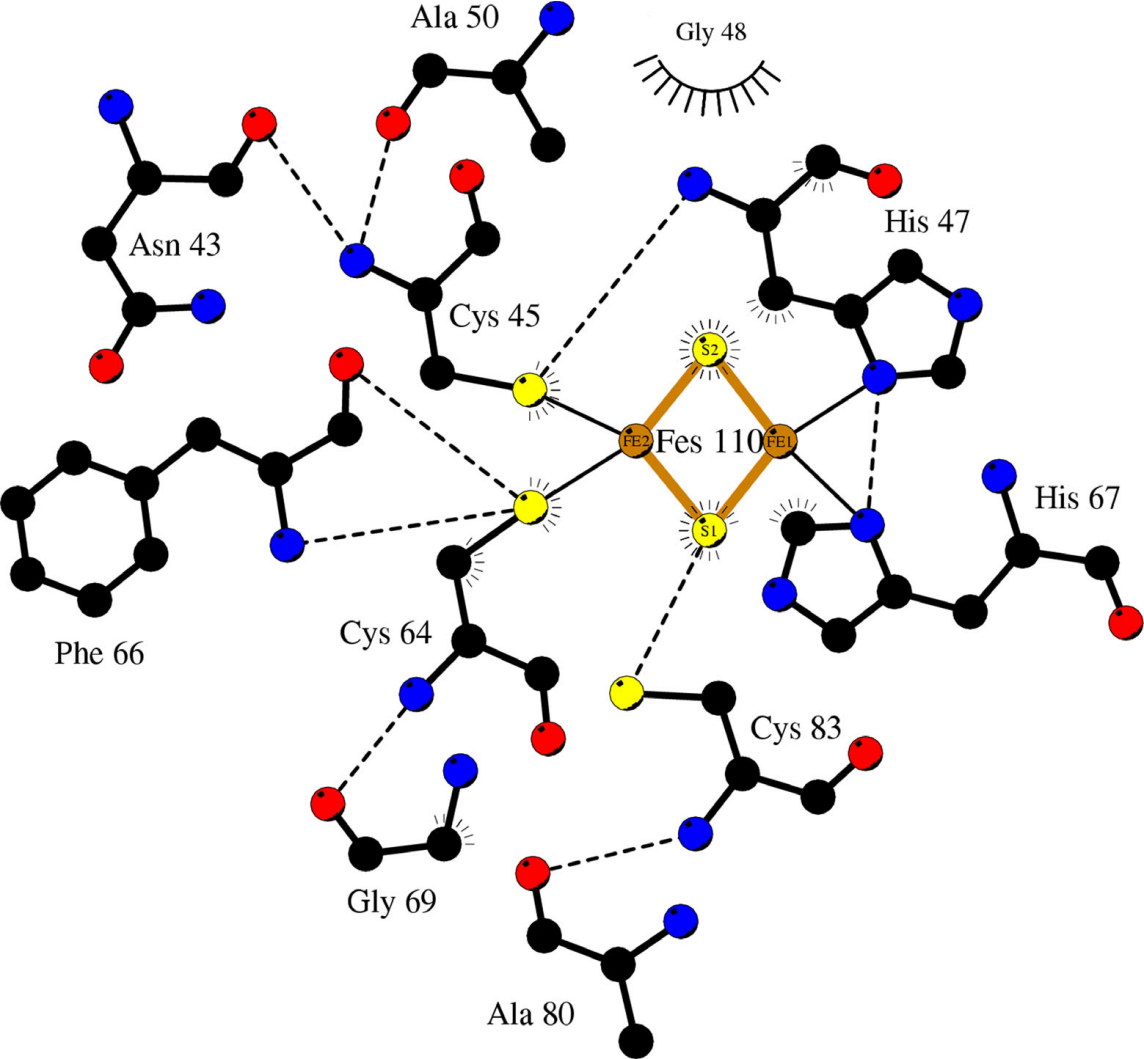
**Residues coordinating Rieske [2Fe-2S] cluster in BPDO-F_B1_**. All residues interacting with Rieske iron-sulfur cluster or its ligands. Hydrogen bonds are displayed in dashed lines. Gly-48 interacts with His-47 and Ala-50 via hydrophobic interactions. Note the interaction between the side-chain of Cys-83 and the iron-sulfur cluster, a feature not present in other known Rieske oxygenase ferredoxins.

The reduction potential of the Rieske cluster in bacterial dioxygenase systems is expected to be approximately -150 mV, similar to that found in BPDO-F_LB400 _[47] and NDO-F_9816-4 _(Lindsay Eltis, personal communicaton). It is believed that the local electrostatic environment, created by charged and hydrogen bonded residues near the cluster, differentiates the reduction potential of the cluster from homologous structures. Thus the low potential oxygenase ferredoxins, with negative reduction potentials, have an electrostatic environment that is more negative than the mitochondrial Rieske ferredoxins, with positive reduction potentials. BPDO-F_B1 _is the ideal protein for exploring these effects; unlike the other Rieske oxygenase ferredoxins, this protein contains a residue which hydrogen bonds to the cluster through the side-chain (Figure 6), as opposed to through the main-chain as present in other ferredoxins. Thus substitution of Cys-83 with alanine, valine, or serine, can probe the effect of local charge and hydrogen bonding on the reduction potential. Interestingly, the conserved Phe-71 in dioxygenase Rieske ferredoxins is exclusively a tyrosine in the Rieske ferredoxins found in mitochondrial and chloroplast electron transport chains. The increased polarity or hydrogen bonding ability of the tyrosine may assist in raising the reduction potential of these ferredoxins. This provides a second rational target for mutational analysis.

### Comparison of BPDO-O_B1 _to other Rieske oxygenases

**Figure 7:**
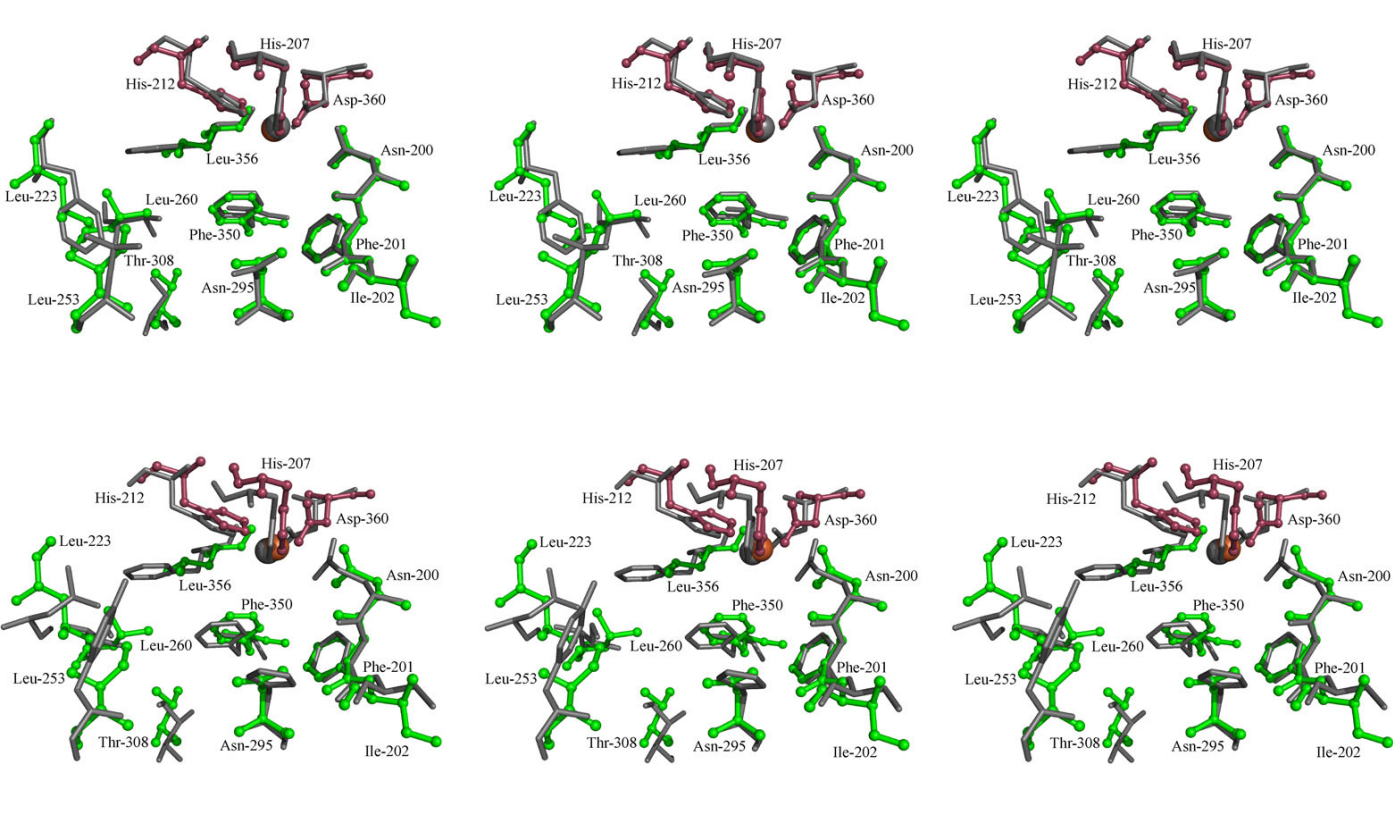
**Comparison of biphenyl 2,3-dioxygenase active site to other Rieske oxygenaes**. Comparison of the active sites of NDO-O_9816-4 _and BPDO-O_RHA1 _with BPDO-O_B1_. NDO-O_9816-4 _(top) and BPDO-O_RHA1 _(bottom) active site residues are shown as smooth grey lines and the active site iron is shown as a grey sphere. BPDO-O_B1 _is aligned and overlaid in each of the images. Residues that coordinate the mononuclear iron are shown as red ball and stick models, while residues that form the active site pocket are shown as green ball and stick models. The mononuclear iron for BPDO-O_B1 _is shown as a rust colored sphere. Residues are labeled using numbering from BPDO-O_B1_. Left and center images are wall-eyed stereo, center and right images are cross-eyed stereo.

The X-ray diffraction structural model shows that BPDO-O_B1 _is structurally similar to other known ROs, as predicted by sequence analysis. Structure and sequence alignment confirm that NDO-O_9816-4 _is the most structurally similar, with most of the variation in the α subunit around the active site. Table 2 gives information on sequence and structural similarity between BPDO-O_B1 _and other ROs. Of the 21 residues that form the active site, only 6 differ from NDO-O_9816-4_. Thr-308 in BPDO-O_B1_, analogous to Ser-310 in NDO-O_9816-4_, is positioned at the entrance to the active site. Thr-308 seems to push residue Leu-260 toward the entrance of the active site compared to the position of Val-260 in NDO-O_9816-4_. This change results in an indentation in the wall at the far side of the active site. Leu-356 in BPDO-O_B1_, analogous to Trp-358 in NDO-O_9816-4_, also contributes to this relative indentation in the active site wall. Residue Phe-224 in NDO-O_9816-4 _is also in this bulge region and is analogous to Leu-223 in BPDO-O_B1_. Leu-226 is conserved between BPDO-O_B1 _and NDO-O_9816-4_, but the side-chain positions differ. These mutations and side-chain rotamer conformations effectively make a hydrophobic indentation in the wall of the active site, both changing the shape and increasing the volume of the active site compared to NDO-O_9816-4_. A similar indentation is present in the structure of BPDO-O_RHA1 _[16] (Figure 7). In both BPDO-O_RHA1 _and BPDO-O_B1_, the phenyl ring of biphenyl distal to the mononuclear iron is in this portion of the active site. By situating the distal ring in this location, the enzyme is able to position the 2 and 3 carbons to be directly facing the mononuclear iron. In NDO-O_9816_, the side-chain of Trp-358 occupies part of this indentation, which would cause the biphenyl substrate to rotate slightly, relative to the mononuclear iron. This rotation would move the 2 carbon farther away from the iron and the 4 carbon closer. This would also explain the differences in the regioselectivity of enzymes, where BPDO-O_B1 _and BPDO-O_RHA1 _produce the *cis*-2,3-biphenyldiol exclusively, while NDO-O_9816-4 _produces a mixture of the *cis*-2,3- and *cis*-3,4-biphenyldiol [51].

The BPDO-O_RHA1 _(PDB entry 1ULI) active site has a significantly smaller volume, ~27 Å^3^, than BPDO-O_B1_, ~43 Å^3^. This is due to bulkier side-chains in BPDO-O_RHA1 _and variability in the loop positions at the active site entrance. Comparing the ligand bound structures, biphenyl carbons 2 and 3 are closer to the mononuclear iron in BPDO-O_RHA1_, 4.3 and 4.6 Å respectively, as compared to BPDO-O_B1 _which has an average distance of approximately 5.0 Å for both the 2 and 3 carbons in the three subunits. However, in both BPDO-O_RHA1 _and BPDO-O_B1_, the distances between the biphenyl and the iron-bound water are similar. The closer position in BPDO-O_RHA1 _is mediated by an interaction with Met-222, which is not present in BPDO-O_B1_. The glycine (Gly-205) present in the structurally analogous position leaves room for the ligand to move slightly further from the iron. The lack of large side-chain rearrangement upon substrate binding is similar to results seen in NDO-O_9816-4 _[27,40] and BPDO-O_RHA1 _[16]. The loop covering the active site entrance of BPDO-O_B1 _shifts upon binding of biphenyl. While large changes in this loop are not seen in the structures of NDO-O_9816-4_, this loop is believed to be flexible in NDO-O_9816-4_[27,40]. BPDO-O_RHA1 _does show significant movement of residues 271 – 276 shifting 1 – 2 Å toward the active site, with the side-chain Leu-274 forming van der Waals interactions with the ligand [16]. Loops covering the active site in the α_3 _Rieske monooxygenase 2-oxoquinoline 8-monooxygenase also demonstrate changes upon substrate binding [30]. This loop motion observed in the BPDO-O_B1 _and other RO structures allows the active site to "breathe" to accommodate ligands and may be one of the key features that allows this class of enzymes to perform catalysis on diverse sets of substrates with respect to overall size and shape.

### Role of oxygenase active site entrance

**Figure 8:**
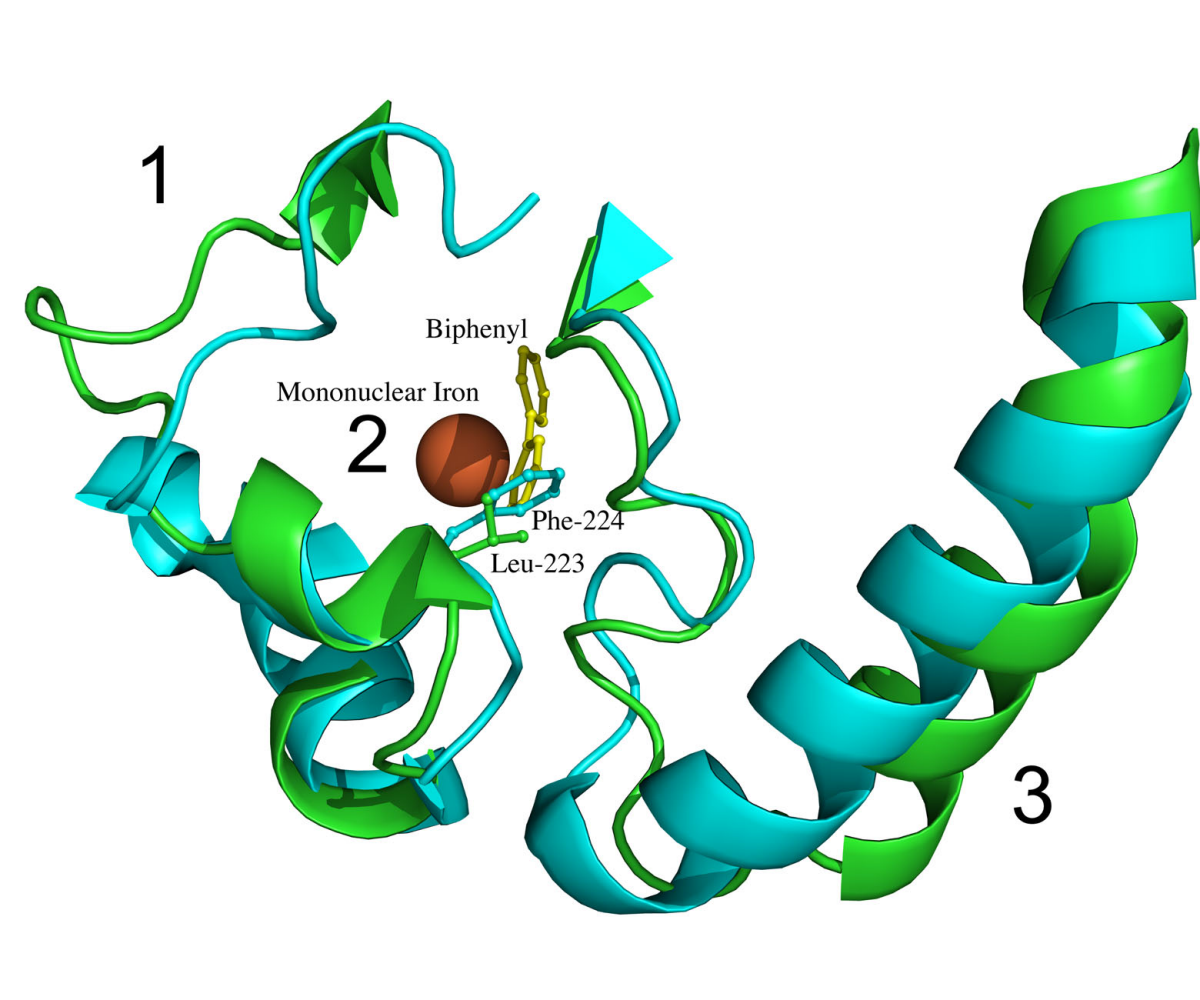
**Comparison of the active site entrances of BPDO-O_B1 _and NDO-O_9816-4_**. Cartoon diagrams of the active site entrances for BPDO-O_B1 _(green) and NDO-O_9816-4 _(cyan). Multiple differences in the loop structure lead to a larger opening to the active site in BPDO-O_B1_. At position 1, the loop structure is pushed away from the entrance, compared to NDO-O_9816-4_. Position 2 shows the increased bulk of the Phe-224 side chain in the entrance opening, compared to the side chain of Leu-223 of BPDO-O_B1_. Finally, postion 3 shows a shift of the helix, which pulls the active site entrance open, compared to NDO-O_9816-4_. Biphenyl bound in the active site is shown in yellow and the mononuclear iron is shown as a rust-colored sphere. See Additional file 1, which shows these structures rotating in three dimensional space.

Evidence for large aromatic compound dihydroxylation by NDO-O_9816-4 _has not been observed in the past. While the overall active site volumes are relatively similar between NDO-O_9816-4 _and BPDO-O_B1_, the active site entrance of BPDO-O_B1 _is larger. Phe-235 is positioned further away from the active site in BPDO-O_B1 _compared to the analogous residue in NDO-O_9816-4_. This, along with the decreased side-chain bulk at Leu-223, compared to NDO-O_9816-4_, effectively increases the size of the active site entrance in BPDO-O_B1_. Figure 8 and Additional file 1 demonstrate the differences in the active site entrance between BPDO-O_B1 _and NDO-O_9816-4_. The entrance to RO active sites is similar to an inverted funnel, with a small aperture leading to a large vestibule. Based on structural comparison, we propose that the shape and size of the active site entrance may keep larger substrates out of the NDO-O_9816-4 _active site even though there would be enough space inside the pocket to accommodate the ligand. Drawing from that comparison, we also believe that the active site entrance could influence the rates of product turnover by BPDO-O_B1 _with respect to large compounds, such as benzo[*a*]pyrene and benzo[*a*]anthracene. While studies to determine rates of product formation by BPDO-O_B1 _have not been performed to date, it has been shown that biotransformation of large compounds, such as benzo[*a*]pyrene and benzo[*a*]anthracene are much less efficient than those for smaller compounds such as naphthalene, and biphenyl [2,3,8,11,52]

## Conclusion

Crystal structures of BPDO-F_B1 _and BPDO-O_B1 _from *Sphingobium yanoikuyae *strain B1 are presented and demonstrate strong structural conservation with other RO ferredoxin and oxygenase components. The structures reported here provide a rational basis for the ability of BPDO-O_B1 _to catalyze large aromatic substrates. It also provides a framework to interpret the product regioselectivity of BPDO-O_B1 _and the differences in product regioselectivity compared to other ROs. While the Rieske ferredoxin structure is very similar to other Rieske ferredoxins, the differences in amino acid composition near the cluster in BPDO-F_B1 _provide a unique opportunity among the Rieske oxygenase ferredoxins to examine the effect of cluster environment and hydrogen bonding on reduction potential.

## Methods

### Protein expression, purification and crystallization

BPDO-O_B1_, bphA1fA2f [11,14] and BPDO-F_B1_, bphA4 [10], were each cloned from B1 into the protein expression vectors pET101D (Invitrogen, Carlsbad, CA) and pT7-7, respectively, and expressed as described in Yu et al. [45]. Crystallization of the ferredoxin protein was performed using 33 mg/mL of BPDO-F_B1 _protein in a 50 mM phosphate buffer, pH 6.8, with 0.1 M citric acid and 1.6 M ammonium sulfate as precipitants. Crystallization of the oxygenase protein was performed using purified BPDO-O_B1 _protein at 20 mg/ml in a 20 mM potassium phosphate buffer, pH 6.8 [45]. Mineral oil was used as a cryoprotectant for the crystals.

Oxygenase crystals were also used for soaking experiments in an attempt to generate the BPDO-O_B1 _protein-biphenyl complex. Soaking experiments were based on similar experiments previously done with NDO-O_9816-4 _to generate crystals of ligand-bound enzyme [27,38,40]. Crystals were moved to fresh drops containing reservoir buffer. Ethanol saturated with biphenyl was added to these drops. The crystals tolerated up to 5% ethanol in the drop. Crystals were allowed to soak for 3–5 days, then were removed from the drop and flash-cooled to 100 K. Mineral oil was used as a cryoprotectant.

### Data collection, processing, structure solution and refinement

X-ray diffraction data for BPDO-F_B1 _were collected on beamline X6A at Brookhaven National Laboratory and data for BPDO-O_B1 _were collected on the IMCA-CAT beamline 17-ID at the Advanced Photon Source in Argonne National Laboratory. Crystallographic statistics are presented in Table 1. d*TREK [53] was used to process the data for BPDO-F_B1 _to a resolution of 1.60Å. Analyzing systematic absences, the space group was determined to be either P6_1_22 or P6_5_22. Molecular replacement using AMoRe [54] and a polyalanine model based on BPDO-F_LB400 _(PDB entry 1FQT) produced a solution in space group P6_5_22. Model building using O [55] and Coot [56], density modification using DM, and refinement using Refmac5 [57] from the CCP4-4.0 program suite were assisted by non-crystallographic symmetry between the two monomers in the asymmetric unit. These NCS restraints were loosened as refinement progressed. Cycles of Refmac5 with ARP/wARP [58,59] or the Coot find waters routines were used to identify solvent atoms. Asp-96 in chain B was modeled as having two side chain conformations. A single round of TLS optimization was used at the end of refinement with each protein monomer acting as a TLS group.

Data collected from native BPDO-O_B1 _crystals was processed using d*TREK [53] to a resolution of 1.70 Å. Analysis of systematic absences suggested that the space group was P3_x_21. Molecular replacement was performed using a polyalanine model based on NDO-O_9816-4_(PDB entry 1NDO) [28]. Molecular replacement using AMoRe [54] gave a clear solution in the space group P3_1_21. Initial refinement of the polyalanine model with the program Refmac5 [57] of the CCP4-5.0.2 [60] suite of programs yielded good starting electron densities. The molecular visualization program O [55] was used for model building. After the bulk of the structure was modeled, refinement was continued with Refmac5 without NCS restraints. Solvent molecules were found using the program Arp/Warp [58,59] and multiple side-chain conformations were modeled using the molecular visualization programs XtalView [61] and Coot [56]. The calculated solvent content was 50 % [62]. The asymmetric unit contains a complete α_3_β_3 _protein.

Data from biphenyl soaked oxygenase crystals were processed using d*TREK to a resolution of 2.8 Å. The native BPDO-O_B1 _structure, with solvent molecules removed, was used as a starting point for the refinement of the ligand bound structure. An energy-minimized structure of the small molecule biphenyl was constructed using the program SYBYL 7.1 [63]. This model was used to create a refinement dictionary for Refmac5 using the ligand sketcher program in CCP4-5.0.2. After initial refinement of the protein model, the biphenyl ligand was modeled into the active site of the enzyme where electron density maps showed un-modeled density in both 2Fo-Fc and Fo-Fc maps. The torsion angle between the two rings of biphenyl was allowed to rotate during refinement and solvent molecules were modeled as appropriate.

### Sequence and structural alignments

TCoffee [64] was used to produce a structure-based sequence alignment of the four known RO ferredoxin structures and 19 other RO ferredoxin sequences. Structural alignment of native BPDO-O_B1 _with structures of other RO oxygenases was performed using the program Indonesia [65]. Structural alignments in Indonesia were done pairwise using the Levitt and Gerstein method with a cut-off value of 3.5 Å. Sequence alignment and comparison was done using full sequences for each of the proteins without structural information. Protein-protein sequence alignments were performed using blastp on the NCBI blast server [66]. All figures showing structures were created using PyMOL 0.98 [67]. Figure 6 was produced using a modified version of LigPlot [68].

## Abbreviations

RO – Rieske Oxygenase

BPDO-O_B1 _– Biphenyl 2,3-dioxygenase from *Sphingobium yanoikuyae *strain B1

BPDO-F_B1 _– Biphenyl 2,3-dioxygenase ferredoxin from *Sphingobium yanoikuyae *strain B1

BPDO-O_RHA1 _– Biphenyl 2,3-dioxygenase from *Rhodococcus *sp. strain RHA1

NDO-F_9816-4 _– Naphthalene 1,2-dioxygenase ferredoxin from *Pseudomonas *sp. strain NCIB 9816-4

NDO-O_9816-4 _– Naphthalene 1,2-dioxygenase from *Pseudomonas *sp. strain NCIB 9816-4

B1 – *Sphingobium yanoikuyae *strain B1

## Authors' contributions

D.J.F. generated the biphenyl-bound oxygenase crystals and carried out the structural work with the oxygenase component. E.N.B. carried out work on the ferredoxin component. D.J.F. and E.N.B. contributed equally to the drafting of this manuscript. C.-L. Y. expressed, purified and crystallized both the ferredoxin and oxygenase components. R.E.P. and D.T.G. conceived of the studies and gave guidance. S.R. was involved in planning X-ray crystallographic experiments and the interpretation of results. All authors have read and approved the manuscript.

## Supplementary Material

Additional File 1 Comparison of the active site entrances of BPDO-OB1 and NDO-O9816-4. Animation of cartoon diagrams of the active site entrances for BPDO-O_B1 _(green) and NDO-O_9816-4 _(cyan). Multiple differences in the loop structure lead to a larger opening to the active site in BPDO-O_B1_. Biphenyl bound in the active site is shown in yellow and the mononuclear iron is shown as a rust-colored sphere. Phe-224 (NDO-O_9816-4_) and Leu-223 (BPDO-O_B1_) are shown with side chains as ball and stick models. Click here for file

## Figures and Tables

**Table 1:** Summary of crystallographic data and refinement statistics

**Crystal**	**Ferredoxin (2I7F)**	**Oxygenase (native) (2GBW)**	**Oxygenase (biphenyl bound) (2GBX)**
Space group	P6_5_22	P3_1_21	P3_1_21
Cell parameters			
a = b (Å)	62.064	134.96	134.43
c (Å)	238.436	219.89	220.53
Resolution (Å)^1^	9.49 – 1.90 Å (1.96 – 1.90 Å)	19.80 -1.70 Å (1.76 -1.70 Å)	43.15 – 2.75 (2.85 – 2.75)
Data collection			
X-ray source	NSLS X6A	IMCA-CAT 17-ID	IMCA-CAT 17-ID
Wavelength (Å)	1.03320	1.00000 Å	1.00000 Å
Total observations	108720	1083097	343108
Unique observations	20866	241600	56040
Completeness (%)^1^	93.7 % (65.2 %)	95.3 % (98.1 %)	92.6 % (99.7 %)
(I)/σ(I)^1^	8.2 (2.9)	8.2 (2.9)	9.6 (3.7)
Multiplicity^1^	5.21 (3.46)	4.48 (4.10)	6.12 (7.14)
R_sym_^1,2^	0.121 (0.417)	0.083 (0.394)	0.104 (0.409)
Crystallographic refinement			
R_factor_/R_free _(%)^3^	19.1/20.3	18.8/22.9	22.8/26.9
RMSD from ideality			
Bond lengths (Å)	0.017 Å	0.017 Å	0.006 Å
Bond angles (deg)	1.925°	1.616°	0.880°

^1 ^Outer shell values are in parentheses.

^2 ^Rsym=∑hkl∑i|Ii(hkl)−I(hkl)|∑hkl∑i|Ii(hkl)|
 MathType@MTEF@5@5@+=feaafiart1ev1aaatCvAUfKttLearuWrP9MDH5MBPbIqV92AaeXatLxBI9gBaebbnrfifHhDYfgasaacH8akY=wiFfYdH8Gipec8Eeeu0xXdbba9frFj0=OqFfea0dXdd9vqai=hGuQ8kuc9pgc9s8qqaq=dirpe0xb9q8qiLsFr0=vr0=vr0dc8meaabaqaciaacaGaaeqabaqabeGadaaakeaacqWGsbGudaWgaaWcbaGaem4CamNaemyEaKNaemyBa0gabeaakiabg2da9maalaaabaWaaabeaeaadaaeqaqaamaaemaabaGaemysaK0aaSbaaSqaaiabdMgaPbqabaGcdaqadaqaaiabdIgaOjabdUgaRjabdYgaSbGaayjkaiaawMcaaiabgkHiTiabdMeajnaabmaabaGaemiAaGMaem4AaSMaemiBaWgacaGLOaGaayzkaaaacaGLhWUaayjcSdaaleaacqWGPbqAaeqaniabggHiLdaaleaacqWGObaAcqWGRbWAcqWGSbaBaeqaniabggHiLdaakeaadaaeqaqaamaaqababaWaaqWaaeaacqWGjbqsdaWgaaWcbaGaemyAaKgabeaakmaabmaabaGaemiAaGMaem4AaSMaemiBaWgacaGLOaGaayzkaaaacaGLhWUaayjcSdaaleaacqWGPbqAaeqaniabggHiLdaaleaacqWGObaAcqWGRbWAcqWGSbaBaeqaniabggHiLdaaaaaa@647A@, where I_i_(hkl) is the *i*th measurement of reflection hkl and I(hkl) is the average for that reflection.

^3 ^Rfactor=∑hkl||Fobs|−k|Fcalc||∑hkl|Fobs|
 MathType@MTEF@5@5@+=feaafiart1ev1aaatCvAUfKttLearuWrP9MDH5MBPbIqV92AaeXatLxBI9gBaebbnrfifHhDYfgasaacH8akY=wiFfYdH8Gipec8Eeeu0xXdbba9frFj0=OqFfea0dXdd9vqai=hGuQ8kuc9pgc9s8qqaq=dirpe0xb9q8qiLsFr0=vr0=vr0dc8meaabaqaciaacaGaaeqabaqabeGadaaakeaacqWGsbGudaWgaaWcbaGaemOzayMaemyyaeMaem4yamMaemiDaqNaem4Ba8MaemOCaihabeaakiabg2da9maalaaabaWaaabeaeaadaabdaqaamaaemaabaGaemOray0aaSbaaSqaaiabd+gaVjabdkgaIjabdohaZbqabaaakiaawEa7caGLiWoacqGHsislcqWGRbWAdaabdaqaaiabdAeagnaaBaaaleaacqWGJbWycqWGHbqycqWGSbaBcqWGJbWyaeqaaaGccaGLhWUaayjcSdaacaGLhWUaayjcSdaaleaacqWGObaAcqWGRbWAcqWGSbaBaeqaniabggHiLdaakeaadaaeqaqaamaaemaabaGaemOray0aaSbaaSqaaiabd+gaVjabdkgaIjabdohaZbqabaaakiaawEa7caGLiWoaaSqaaiabdIgaOjabdUgaRjabdYgaSbqab0GaeyyeIuoaaaaaaa@63A0@, where F_obs _and F_calc _are the observed and calculated structure factors, respectively. R_free _is the same, but for a test set of reflections not used in refinement.

**Table 2:** Sequence and structural statistics for various Rieske oxygenases compared to biphenyl 2,3-dioxygenase

**Ferredoxin (PDB ID)**	**RMSD (Cα)**	**Sequence identity**	**Sequence similarity**
BPDO-F_LB400 _(1FQT)	1.017 (97)	36.6 %	56.4 %
T4MOC-F (1SJG)	1.697 (93)	24.1 %	52.7 %
CARDO-F_CA10 _(1VCK)	1.312 (98)	36.3 %	60.8 %

**Terminal oxygenase α Subunit (PDB ID)**	**RMSD (Cα)**	**Sequence identity**	**Sequence similarity**

NDO-O_9816-4 _(1NDO)	1.24 (420)	43.5 %	62.0 %
NBDO-O_JS765 _(2BMO)	1.23 (413)	41.2 %	59.4 %
BPDO-O_RHA1 _(1ULI)	1.48 (388)	39.6 %	55.1 %
CDO-O_IP01 _(1WQL)	1.50 (377)	36.8 %	52.7 %
NDO-O_12038 _(2B1X)	1.59 (358)	35.0 %	52.5 %

**Terminal oxygenase β Subunit (PDB ID)**	**RMSD (Cα)**	**Sequence identity**	**Sequence similarity**

NDO-O_9816-4 _(1NDO)	1.19 (156)	28.1 %	46.0 %
NBDO-O_JS765 _(2BMO)	1.19 (160)	29.5 %	47.0 %
BPDO-O_RHA1 _(1ULI)	1.15 (159)	31.6 %	53.2 %
CDO-O_IP01 _(1WQL)	1.17 (161)	31.8 %	48.6 %
NDO-O_12038 _(2B1X)	1.20 (156)	35.3 %	49.3 %

**Table 3:** Structurally analogous residues near the iron-sulfur cluster in various Rieske dioxygenase ferredoxins

**BPDO-F_B1_**	**BPDO-F_LB400_**	**T4MOC-F**	**CARDO-F_CA10_**
*Rieske cluster coordinating residues*			

CYS 45	CYS 43	CYS 45	CYS 46
HIS 47	HIS 45	HIS 47	HIS 48
CYS 64	CYS 63	CYS 64	CYS 65
HIS 67	HIS 66	HIS 67	HIS 68

*Outer shell residues*			

ASN 43	ASP 41	ALA 43	ASP 44
GLY 48	GLY 46	GLN 48	GLY 49
ALA 50	TRP 48		ALA 51
PHE 66	LEU 65	ALA 66	PHE 67
GLY 69	GLY 68	TRP 49*	GLY 69
ALA 80	SER 79	ASN 80	SER 81
CYS 83	PRO 82		CYS 84

In the structural superposition, residues marked with an asterisk were more than 3.0 Å from the corresponding residue in BPDO-O_B1_.

## References

[B1] Khan AA, Wang RF, Cao WW, Franklin W, Cerniglia CE (1996). Reclassification of a polycyclic aromatic hydrocarbon-metabolizing bacterium, *Beijerinckia* sp. strain B1, as *Sphingomonas yanoikuyae* by fatty acid analysis, protein pattern analysis, DNA-DNA hybridization, and 16S ribosomal DNA sequencing. Int J Syst Bacteriol.

[B2] Gibson DT, Roberts RL, Wells MC, Kobal VM (1973). Oxidation of biphenyl by a *Beijerinckia* species. Biochem Biophys Res Commun.

[B3] Gibson DT (1999). Beijerinckia sp strain B1: a strain by any other name. J Ind Microbiol Biotechnol.

[B4] Kim SJ, Kweon O, Freeman JP, Jones RC, Adjei MD, Jhoo JW, Edmondson RD, Cerniglia CE (2006). Molecular cloning and expression of genes encoding a novel dioxygenase involved in low- and high-molecular-weight polycyclic aromatic hydrocarbon degradation in *Mycobacterium vanbaalenii* PYR-1. Appl Environ Microbiol.

[B5] Jouanneau Y, Meyer C (2006). Purification and characterization of an arene *cis*-dihydrodiol dehydrogenase endowed with broad substrate specificity toward polycyclic aromatic hydrocarbon dihydrodiols. Appl Environ Microbiol.

[B6] Demaneche S, Meyer C, Micoud J, Louwagie M, Willison JC, Jouanneau Y (2004). Identification and functional analysis of two aromatic-ring-hydroxylating dioxygenases from a *Sphingomonas* strain that degrades various polycyclic aromatic hydrocarbons. Appl Environ Microbiol.

[B7] Jouanneau Y, Meyer C, Jakoncic J, Stojanoff V, Gaillard J (2006). Characterization of a naphthalene dioxygenase endowed with an exceptionally broad substrate specificity toward polycyclic aromatic hydrocarbons. Biochemistry.

[B8] Gibson DT, Mahadevan V, Jerina DM, Yogi H, Yeh HJ (1975). Oxidation of the carcinogens benzo[*a*]pyrene and benzo[*a*]anthracene to dihydrodiols by a bacterium. Science.

[B9] Zylstra GJ, Kim E (1997). Aromatic hydrocarbon degradation by *Sphingomonas yanoikuyae* B1. J Ind Microbiol Biotechnol.

[B10] Kim E, Zylstra GJ (1999). Functional analysis of genes involved in biphenyl, naphthalene, phenanthrene, and *m*-xylene degradation by *Sphingomonas yanoikuyae* B1. J Ind Microbiol Biotechnol.

[B11] Yu CL, Liu W, Ferraro DJ, Brown EN, Ramaswamy S, Parales RE, Gibson DT (2007). Purification, characterization, and crystallization of the components of a biphenyl dioxygenase system from* Sphingobium yanoikuyae* B1. J Ind Microbiol Biotechnol.

[B12] Cho O, Choi KY, Zylstra GJ, Kim YS, Kim SK, Lee JH, Sohn HY, Kwon GS, Kim YM, Kim E (2005). Catabolic role of a three-component salicylate oxygenase from *Sphingomonas yanoikuyae* B1 in polycyclic aromatic hydrocarbon degradation. Biochem Biophys Res Commun.

[B13] Bae M, Kim E (2000). Association of a common reductase with multiple aromatic terminal dioxygenases in *Sphingomonas yanoikuyae* strain B1. J Microbiol.

[B14] Moritz E, Ni´ Chadhain SM, Kim E, Zylstra GJ (2004). Cloning, sequencing, and characterization of a multicomponent biphenyl dioxygenase system from *Sphingomonas yanoikuyae* B1: Abstract. 104th General Meeting of the American Society for Microbiology, New Orleans, LA.

[B15] Dong X, Fushinobu S, Fukuda E, Terada T, Nakamura S, Shimizu K, Nojiri H, Omori T, Shoun H, Wakagi T (2005). Crystal structure of the terminal oxygenase component of cumene dioxygenase from *Pseudomonas fluorescens* IP01. J Bacteriol.

[B16] Furusawa Y, Nagarajan V, Tanokura M, Masai E, Fukuda M, Senda T (2004). Crystal structure of the terminal oxygenase component of biphenyl dioxygenase derived from *Rhodococcus* sp. strain RHA1. J Mol Biol.

[B17] Imbeault NY, Powlowski JB, Colbert CL, Bolin JT, Eltis LD (2000). Steady-state kinetic characterization and crystallization of a polychlorinated biphenyl-transforming dioxygenase. J Biol Chem.

[B18] Zielinski M, Kahl S, Hecht HJ, Hofer B (2003). Pinpointing biphenyl dioxygenase residues that are crucial for substrate interaction. J Bacteriol.

[B19] Furukawa K (2000). Engineering dioxygenases for efficient degradation of environmental pollutants. Curr Opin Biotechnol.

[B20] Furukawa K, Suenaga H, Goto M (2004). Biphenyl dioxygenases: functional versatilities and directed evolution. J Bacteriol.

[B21] Brenner V, Arensdorf JJ, Focht DD (1994). Genetic construction of PCB degraders. Biodegradation.

[B22] Pieper DH (2005). Aerobic degradation of polychlorinated biphenyls. Appl Microbiol Biotechnol.

[B23] Ferraro DJ, Gakhar L, Ramaswamy S (2005). Rieske business: Structure-function of Rieske non-heme oxygenases. Biochem Biophys Res Commun.

[B24] Lee K (1998). Involvement of electrostatic interactions between the components of toluene dioxygenase from *Pseudomonas putida* F1. J Microbiol Biotechnol.

[B25] Kabsch W (1976). A solution for the best rotation to relate two sets of vectors. Acta Crystallogr A.

[B26] Gakhar L, Malik ZA, Allen CC, Lipscomb DA, Larkin MJ, Ramaswamy S (2005). Structure and increased thermostability of *Rhodococcus* sp. naphthalene 1,2-dioxygenase. J Bacteriol.

[B27] Karlsson A, Parales JV, Parales RE, Gibson DT, Eklund H, Ramaswamy S (2003). Crystal structure of naphthalene dioxygenase: side-on binding of dioxygen to iron. Science.

[B28] Kauppi B, Lee K, Carredano E, Parales RE, Gibson DT, Eklund H, Ramaswamy S (1998). Structure of an aromatic-ring-hydroxylating dioxygenase-naphthalene 1,2-dioxygenase. Structure.

[B29] Nojiri H, Ashikawa Y, Noguchi H, Nam JW, Urata M, Fujimoto Z, Uchimura H, Terada T, Nakamura S, Shimizu K, Yoshida T, Habe H, Omori T (2005). Structure of the terminal oxygenase component of angular dioxygenase, carbazole 1,9a-dioxygenase. J Mol Biol.

[B30] Martins BM, Svetlitchnaia T, Dobbek H (2005). 2-Oxoquinoline 8-monooxygenase oxygenase component: active site modulation by Rieske-[2Fe-2S] center oxidation/reduction. Structure.

[B31] Murzin AG, Brenner SE, Hubbard T, Chothia C (1995). SCOP: a structural classification of proteins database for the investigation of sequences and structures. J Mol Biol.

[B32] Wolfe MD, Altier DJ, Stubna A, Popescu CV, Munck E, Lipscomb JD (2002). Benzoate 1,2-dioxygenase from *Pseudomonas putida*: single turnover kinetics and regulation of a two-component Rieske dioxygenase. Biochemistry.

[B33] Wolfe MD, Lipscomb JD (2003). Hydrogen peroxide-coupled *cis*-diol formation catalyzed by naphthalene 1,2-dioxygenase. J Biol Chem.

[B34] Wolfe MD, Parales J, Lee K, Gibson DT, Lipscomb JD (1999). Substrate binding to the mononuclear ferrous ion of the Rieske dioxygenase naphthalene 1,2-dioxygenase from *Pseudomonas* sp NCIB 9816-4. J Inorg Biochem.

[B35] Wolfe MD, Parales JV, Gibson DT, Lipscomb JD (2001). Single turnover chemistry and regulation of O2 activation by the oxygenase component of naphthalene 1,2-dioxygenase. J Biol Chem.

[B36] Yang TC, Wolfe MD, Neibergall MB, Mekmouche Y, Lipscomb JD, Hoffman BM (2003). Modulation of substrate binding to naphthalene 1,2-dioxygenase by Rieske cluster reduction/oxidation. J Am Chem Soc.

[B37] Yang TC, Wolfe MD, Neibergall MB, Mekmouche Y, Lipscomb JD, Hoffman BM (2003). Substrate binding to NO-ferro-naphthalene 1,2-dioxygenase studied by high-resolution Q-band pulsed 2H-ENDOR spectroscopy. J Am Chem Soc.

[B38] Karlsson A (2002). Structural and mechanistic studies of Rieske dioxygenases. Department of Molecular Biology Uppsala, Sweden, The Swedish University of Agricultural Sciences.

[B39] Karlsson A, Parales JV, Parales RE, Gibson DT, Eklund H, Ramaswamy S (2005). NO binding to naphthalene dioxygenase. J Biol Inorg Chem.

[B40] Carredano E, Karlsson A, Kauppi B, Choudhury D, Parales RE, Parales JV, Lee K, Gibson DT, Eklund H, Ramaswamy S (2000). Substrate binding site of naphthalene 1,2-dioxygenase: functional implications of indole binding. J Mol Biol.

[B41] Friemann R (2005). Structure-function studies of iron sulfur enzyme systems. Department of Molecular Biology Uppsala, Sweden, The Swedish University of Agricultural Sciences.

[B42] Friemann R, Ivkovic-Jensen MM, Lessner DJ, Yu CL, Gibson DT, Parales RE, Eklund H, Ramaswamy S (2005). Structural insight into the dioxygenation of nitroarene compounds: the crystal structure of nitrobenzene dioxygenase. J Mol Biol.

[B43] Parales RE (2003). The role of active-site residues in naphthalene dioxygenase. J Ind Microbiol Biotechnol.

[B44] Ziffer H, Jerina DM, Gibson DT, Kobal VM (1973). Absolute stereochemistry of the (+)-*cis*-1,2-dihydroxy-3-methylcyclohexa-3,5-diene produced from toluene by *Pseudomonas putida*. J Am Chem Soc.

[B45] Ferraro DJ, Okerlund AL, Mowers JC, Ramaswamy S (2006). Structural basis for regioselectivity and stereoselectivity of product formation by naphthalene 1,2-dioxygenase. J Bacteriol.

[B46] Colbert CL, Couture MM, Eltis LD, Bolin JT (2000). A cluster exposed: structure of the Rieske ferredoxin from biphenyl dioxygenase and the redox properties of Rieske Fe-S proteins. Structure.

[B47] Iwata S, Saynovits M, Link TA, Michel H (1996). Structure of a water soluble fragment of the 'Rieske' iron-sulfur protein of the bovine heart mitochondrial cytochrome bc1 complex determined by MAD phasing at 1.5 Å resolution. Structure.

[B48] Nam JW, Noguchi H, Fujimoto Z, Mizuno H, Ashikawa Y, Abo M, Fushinobu S, Kobashi N, Wakagi T, Iwata K, Yoshida T, Habe H, Yamane H, Omori T, Nojiri H (2005). Crystal structure of the ferredoxin component of carbazole 1,9a-dioxygenase of *Pseudomonas resinovorans* strain CA10, a novel Rieske non-heme iron oxygenase system. Proteins.

[B49] Skjeldal L, Peterson FC, Doreleijers JF, Moe LA, Pikus JD, Westler WM, Markley JL, Volkman BF, Fox BG (2004). Solution structure of T4moC, the Rieske ferredoxin component of the toluene 4-monooxygenase complex. J Biol Inorg Chem.

[B50] Parales RE, Lee K, Resnick SM, Jiang H, Lessner DJ, Gibson DT (2000). Substrate specificity of naphthalene dioxygenase: effect of specific amino acids at the active site of the enzyme. J Bacteriol.

[B51] Mahaffey WR, Gibson DT, Cerniglia CE (1988). Bacterial oxidation of chemical carcinogens: formation of polycyclic aromatic acids from benz[a]anthracene. Appl Environ Microbiol.

[B52] Pflugrath JW (1999). The finer things in X-ray diffraction data collection. Acta Crystallogr D Biol Crystallogr.

[B53] Navaza J (1994). AMoRe: an automated package for molecular replacement. Acta Crystallogr A.

[B54] Jones TA, Zou JY, Cowan SW, Kjeldgaard (1991). Improved methods for building protein models in electron density maps and the location of errors in these models. Acta Crystallogr A.

[B55] Emsley P, Cowtan K (2004). Coot: model-building tools for molecular graphics. Acta Crystallogr D Biol Crystallogr.

[B56] Murshudov GN, Vagin AA, Dodson EJ (1997). Refinement of macromolecular structures by the maximum- likelihood method. Acta Crystallogr D Biol Crystallogr.

[B57] Perrakis A, Sixma TK, Wilson KS, Lamzin VS (1997). wARP: improvement and extension of crystallographic phases by weighted averaging of multiple-refined dummy atomic models. Acta Crystallogr D Biol Crystallogr.

[B58] Lamzin VS, Wilson KS (1993). Automated refinement of protein models. Acta Crystallogr D Biol Crystallogr.

[B59] (1994). The CCP4 suite: programs for protein crystallography. Acta Crystallogr D Biol Crystallogr.

[B60] McRee DE (1992). A visual protein crystallographic software system for X11/XView. J Mol Graphics.

[B61] Matthews BW (1968). Solvent content of protein crystals. J Mol Biol.

[B62] SYBYL.

[B63] Poirot O, O'Toole E, Notredame C (2003). Tcoffee@igs: A web server for computing, evaluating and combining multiple sequence alignments. Nucleic Acids Res.

[B64] Madsen D, Johansson P, Kleywegt GJ (2002). Indonesia.

[B65] Altschul SF, Madden TL, Schaffer AA, Zhang J, Zhang Z, Miller W, Lipman DJ (1997). Gapped BLAST and PSI-BLAST: a new generation of protein database search programs. Nucleic Acids Res.

[B66] DeLano WL (2000). The PyMOL Molecular Graphics System.

[B67] Wallace AC, Laskowski RA, Thornton JM (1995). LIGPLOT: a program to generate schematic diagrams of protein-ligand interactions. Protein Eng.

